# *IL*-*6* rs1800795 polymorphism is associated with septic shock-related death in patients who underwent major surgery: a preliminary retrospective study

**DOI:** 10.1186/s13613-017-0247-8

**Published:** 2017-02-28

**Authors:** Maria Angeles Jiménez-Sousa, Luz Maria Medrano, Pilar Liu, Amanda Fernández-Rodríguez, Raquel Almansa, Esther Gomez-Sanchez, Alicia Ortega, María Heredia-Rodríguez, Estefanía Gómez-Pesquera, Eduardo Tamayo, Salvador Resino

**Affiliations:** 1 Unidad de Infección Viral e Inmunidad, Centro Nacional de Microbiología, Instituto de Salud Carlos III (Campus Majadahonda), Carretera Majadahonda- Pozuelo, Km 2.2, 28220 Majadahonda, Madrid Spain; 2 Departamento de Anestesiología y Reanimación, Hospital Clínico Universitario, Valladolid, Spain; 3 Unidad de Investigación Médica en Infección e Inmunidad, Hospital Clínico Universitario-IECSCYL, Valladolid, Spain

**Keywords:** IL-6, SNPs, Septic shock, Survival, Major surgery

## Abstract

**Background:**

Sepsis is a life-threatening organ dysfunction caused by a dysregulated host response to infection, being the primary cause of death from infection, especially if not recognized and treated promptly. The aim of this study was to analyze whether *IL*-*6* rs1800795 polymorphism is associated with septic shock-related death in European white patients who underwent major surgery.

**Methods:**

We performed a retrospective study on 202 septic shock patients who underwent major cardiac or abdominal surgery. The septic shock was established according to the international septic shock definition. The primary outcome variable was the death within 90 days after diagnosis of septic shock. The *IL*-*6* rs1800795 polymorphism was genotyped by Sequenom’s MassARRAY platform.

**Results:**

The median age of the patients was 73 years, 63.4% were male, and more than 40% of patients had heart disease and hypertension. Overall, the survival analysis showed that 111 (55%) patients died with a survival median of 39 days (95% CI 30.7; 47.2). The genetic analysis association with survival was performed under a recessive genetic model (CC vs. GG/CG). Patients with *IL*-*6* rs1800795 CC genotype had higher mortality rate than the *IL*-*6* rs1800795 GG/CG genotype at days 7 [31.6% (6/19) vs. 10.4% (19/183); log-rank test (*p* = 0.005)] and 28 [57.9% (11/19) vs. 33.3% (61/183); log-rank test (*p* = 0.009)], and 90 [68.4% (13/19) vs. 53.5% (98/183); log-rank test (*p* = 0.006)]. The *IL*-*6* rs1800795 CC genotype was associated with higher risk of septic shock-related death during the first 7 days [adjusted hazard ratio (aHR 4.65; *p* = 0.002), 28 days (aHR 2.50; *p* = 0.006), and 90 days (aHR 2.28; *p* = 0.006)] with septic shock. When patients were stratified by type of surgery, those with *IL*-*6* rs1800795 CC genotype who underwent cardiac surgery had higher risk of death during the first 7 days (aHR 18.39; *p* = 0.001) and 28 days (aHR 6.1; *p* = 0.025) than *IL*-*6* rs1800795 GG/GC carrier, whereas patients with *IL*-*6* rs1800795 CC genotype who underwent abdominal surgery had higher risk of death during all follow-up (aHR 1.98; *p* = 0.050) than *IL*-*6* rs1800795 GG/GC carrier.

**Conclusions:**

The presence of *IL*-*6* rs1800795 CC genotype was associated with higher risk of septic shock-related death in patients who underwent major cardiac or abdominal surgery. These findings need robust validation in bigger independent cohorts.

**Electronic supplementary material:**

The online version of this article (doi:10.1186/s13613-017-0247-8) contains supplementary material, which is available to authorized users.

## Background

Sepsis is a life-threatening organ dysfunction caused by a dysregulated host response to infection, being the primary cause of death from infection, especially if it is from an unknown origin and it was not treated promptly [1]. Septic shock is a subset of sepsis in which underlying circulatory and cellular/metabolic abnormalities are profound enough to substantially increase mortality [1]. Despite advances in treatment and supportive care in recent years, septic shock is a major healthcare problem, affecting millions of people around the world each year and killing a high percentage of patients in hospitals [2], especially in intensive care unit (ICU) [2, 3]. In these patients, early and aggressive treatment has not improved survival [4], and patients that survive to sepsis remain at increased risk to death in the following months and years [5].

The immune dysfunction is the main pathophysiological process in septic patients. These patients are severely immunocompromised and are unable to clarify invasive microbial pathogens [6], which may predict the survival of septic patients [7, 8]. Microbial antigens may cause the typical septic inflammatory cascade with overproduction of proinflammatory cytokine (including TNF-α, IL-1, and IL-6), which may enter into the bloodstream causing hypercytokinemia [9]. These proinflammatory cytokines play an important role in the Janus kinase/signal transducer and activator of transcription (JAK–STAT) pathway, by transmitting their information into the cell nucleus for developing a specific response against microbial pathogens [9]. Moreover, proinflammatory cytokines may activate the suppressor of cytokine signaling-3 (SOCS3) and may modulate the cytokine signaling, usually preventing, but in some cases aggravating the outcome of infections [9].

The proinflammatory cytokines may also lead to endothelial damage and intravascular clotting, the formation of blood clots in small blood vessels, multiorgan failure, development of septic shock, and death [9, 10]. Thus, plasma IL-6 levels have been associated with higher risk of sepsis, septic shock, and death [11–14]. However, it is not known clearly why under similar circumstances, some patients eliminate more easily an invading microorganism, whereas other patients develop sepsis and septic shock. In this regard, a variable to consider is the host genetic factor, which has been related to sepsis outcome [15]. On this subject, the rs1800795 single nucleotide polymorphism (SNP) at *IL*-*6* promoter has been related to differential production of IL-6 [16]. Furthermore, the rs1800795 *IL*-*6* polymorphism has been associated with the risk of sepsis and death, but with different results depending on the study [17–21]. Therefore, in patients who underwent major surgery, the role of the *IL*-*6* rs1800795 SNP in septic shock is not clear yet.

The aim of this study was to analyze whether *IL*-*6* rs1800795 polymorphism is associated with septic shock-related death in European patients who underwent major surgery.

## Methods

### Patients

We carried out a retrospective study on 202 European white patients older than 18 who developed septic shock. These patients underwent major cardiac or abdominal surgery at the Hospital Clínico Universitario of Valladolid (Spain) between April 2008 and November 2012. Major surgery was considered as an operative procedure in which the patient was under general anesthesia and respiratory assistance because the patient was not able to breathe independently.

The study was conducted in accordance with the Declaration of Helsinki. All patients gave their written consent for the study. The Ethics Committee of Hospital Clínico Universitario (Valladolid) and Instituto de Salud Carlos III (Majadahonda) approved this study.

### Control groups

We used a control group of 262 patients who only developed systemic inflammatory response syndrome (SIRS) [22] after major surgery (cardiac or abdominal) and had similar age and gender than septic shock patients. These patients were collected at the Hospital Clínico Universitario between 2008 and 2012. Moreover, for healthy subjects, the frequencies of alleles and genotypes for studied polymorphisms were obtained using the 1000 Genomes Project Web site (http://www.1000genomes.org/home), which provide a broad representation of common human genetic variation by applying whole-genome sequencing to a diverse set of individuals from multiple populations [23]. We select the Iberian populations in Spain (IBS) population that included 107 individuals.

### Clinical data

Demographic and clinical data were obtained from medical records: age, gender, type of surgery, prior or pre-existing conditions such as diabetes, chronic obstructive pulmonary disease, hypertension, chronic kidney disease, cancer, liver disease and cardiomyopathy. Cardiopulmonary bypass was carried out in all cardiac surgeries. Acute Physiology and Chronic Health Evaluation (APACHE-II score) [24] and Sequential Organ Failure Assessment (SOFA score) [25] were calculated within the first 24 h after diagnosis, in order to evaluate severity of sepsis.

The diagnosis of septic shock was established according to the criteria laid down by the SCCM/ESICM/ACCP/ATS/SIS International Sepsis Definitions Conference [22]. The presence of infection was either documented or presumed based on clinical findings. In those cases where infection was strongly suspected but not microbiologically confirmed, two experienced clinicians discussed and reached a consensus diagnosis according to physical and laboratory findings.

### DNA genotyping

Total DNA was extracted from peripheral blood with High Pure PCR Template Preparation Kit (Roche Diagnostics GmbH, Mannheim, Germany). DNA samples were sent to the Spanish National Genotyping Center (CeGen; http://www.cegen.org/) for *IL*-*6* rs1800795 polymorphism genotyping by using Sequenom’s MassARRAY platform (San Diego, CA, USA) and the iPLEX^®^ Gold assay design system.

### Outcome variable

The primary outcome variable was the death within 90 days after diagnosis of septic shock. We used three points censoring: 7 days (very early mortality), 28 days (early mortality), and 90 days (late mortality).

### Statistical analysis

For the description of the study population, *p* values were estimated with nonparametric tests: Mann–Whitney *U* test was used for continuous variable and Chi-squared/Fisher’s exact test for categorical variables. For the genetic association study, the survival analysis (Kaplan–Meier and Cox regression analyses) was used to compare the outcome variables according to the presence of *IL*-*6* rs1800795 CC genotype. Data were analyzed using dominant, recessive and additive models, which were tested according to the goodness of fit evaluated by Akaike’s information criterion (AIC) value and Bayesian information criterion (BIC).

Follow-up was censored at 90 days. Survival probabilities were estimated using the Kaplan–Meier product-limit method at 7, 28, and 90 days and compared using the log-rank test. Cox regression analyses were used to investigate the relationship of *IL*-*6* rs1800795 polymorphism with the outcome variable during the first 7, 28, and 90 days. Each Cox regression test was adjusted by the most significant covariables for each outcome variable, avoiding the overfitting of the regression. The covariables were selected by stepwise algorithm in a multivariate model. We included the SNP [enter algorithm (forced entry for the SNP)] and the most relevant characteristics by stepwise algorithm (at each step, factors are considered for removal or entry: a *p* value for entry and exit of 0.15 and 0.20, respectively). The covariables used were APACHE-II score, gender, age, antibiotic treatment, type of surgery (cardiac or abdominal), elective surgery (emergency or scheduled), peritonitis, comorbidities [obesity, diabetes heart disease, chronic obstructive pulmonary disease (COPD)], hypertension, neoplasia, liver disease, smoker, and high alcohol intake. All statistical analyses were performed by using the IBM SPSS Statistics for Windows, version 21.0 (IBM Corp, Chicago, Armonk, NY, USA).

In addition, Hardy–Weinberg equilibrium (HWE) analyses were computed by Haploview 4.2 software, considering equilibrium when *p* > 0.05.

## Results

### Characteristics of the study population

Table 1 shows demographic and clinical characteristics of shock septic patients at the time of septic shock diagnosis according to the *IL*-*6* rs1800795 genotype. Overall, the median age was 73 years, 63.4% were male, and more than 40% of patients had heart disease and hypertension. Regarding the type of surgery, 40.1% were cardiac surgery and 63.4% were emergency surgery. The most commonly isolated pathogens were gram-negatives (52.5%), and more than 40% of patients had peritonitis or pneumonia. When the population was stratified according to *IL*-*6* rs1800795 genotype, we only found significant differences in COPD frequencies (*p* = 0.027).

**Table 1:** Summary of epidemiological and clinical characteristics of septic shock patients who underwent major surgery

Characteristics	All patients	*IL*-*6* rs1800795 polymorphism		
		GG/CG	CC	*p* value*
No. of patients	202	183	19	–
Gender (male)	128 (63.4%)	115 (62.8%)	13 (68.4%)	0.631
Age (years)	73 (17)	73 (17)	73 (17)	0.974
Prior or pre-existing conditions				
Smoker	36 (17.8%)	35 (19.1%)	1 (5.3%)	0.207
Alcoholism	15 (7.4%)	14 (7.7%)	1 (5.3%)	0.999
Obesity	30 (14.9%)	27 (14.8%)	3 (15.8%)	0.999
Diabetes	26 (12.9%)	23 (12.6%)	3 (15.8%)	0.718
Heart disease	95 (45.5%)	82 (44.8%)	10 (52.6%)	0.515
Chronic obstructive pulmonary disease	35 (17.3%)	28 (15.3%)	7 (36.8%)	*0.027*
Hypertension	111 (50.0%)	100 (54.6%)	11 (57.9%)	0.789
Chronic kidney disease	30 (14.9%)	28 (15.3%)	2 (10.5%)	0.745
Cancer	47 (23.3%)	41 (22.4%)	6 (31.6%)	0.395
Liver disease	9 (4.5%)	8 (4.4%)	1 (5.3%)	0.597
Surgery				
Cardiac (vs. abdominal)	81 (40.1%)	75 (41%)	6 (31.6%)	0.426
Emergency (vs. scheduled)	128 (63.4%)	116 (63.4%)	12 (63.2%)	0.984
Severity indexes				
SOFA score	9 (4)	9 (4)	9 (3)	0.932
APACHE-II score	16 (5)	17 (5)	15 (8)	0.758
Infection				
Gram-positive	99 (49%)	92 (50.3%)	7 (36.8%)	0.265
Gram-negative	106 (52.5%)	100 (54.6%)	6 (31.6%)	0.055
Fungus	39 (19.3%)	35 (19.1%)	4 (21.1%)	0.767
Catheter bacteraemia	68 (33.7%)	63 (34.4%)	5 (26.3%)	0.476
Surgical site infection	48 (23.8%)	45 (24.6%)	3 (15.8%)	0.573
Urinary tract infection	24 (11.9%)	21 (11.5%)	3 (15.8%)	0.479
Endocarditis	10 (5%)	8 (4.4%)	2 (10.5%)	0.239
Peritonitis	95 (47%)	86 (47%)	9 (47.4%)	0.975
Pneumonia	95 (47%)	89 (48.6%)	6 (31.6%)	0.156

Values are expressed as median (interquartile range) and absolute count (%)

Note that patients may have had more than one organism cultured

*SOFA* Sequential Organ Failure Assessment, *APACHE* Acute Physiology and Chronic Health Evaluation

* *p* values were calculated by Chi-squared test or Fisher’s exact test for categorical variables and Mann–Whitney test for continuous variables. Statistically significant differences are shown in italics

Additional file 1: Table S1 shows demographic and clinical characteristics of shock septic patients stratified by the type of surgery (cardiac and abdominal). The cardiac surgery group had higher frequency of obesity (*p* = 0.045), heart disease (*p* < 0.001), and hypertension (*p* = 0.014) than the abdominal surgery group. Nonetheless, cardiac surgery group had lower frequency of cancer (*p* < 0.001) than the abdominal surgery group. Also, the cardiac surgery group had higher values of SOFA (*p* = 0.030) and APACHE (*p* = 0.032) than the abdominal surgery group. Regarding infection, the cardiac surgery group had higher frequency of catheter bacteraemia (*p* < 0.001), endocarditis (*p* < 0.001), and pneumonia (*p* < 0.001) and lower frequency of peritonitis (*p* < 0.001).

### Characteristics of the *IL*-*6* polymorphism

Table 2 shows the frequencies of *IL*-*6* rs1800795 polymorphism, which displayed missing values <5% and were in HWE (*p* > 0.05). The minor allele frequency was 32%. Data of our cohort were compared to the frequencies of *IL*-*6* rs1800795 polymorphism in SIRS patients and healthy subjects from the IBS [23]. We did not find any significant difference.

**Table 2:** Frequencies of alleles and genotypes for *IL*-*6* rs1800795 polymorphism in septic shock patients compared to Iberian populations in Spain from 1000 Genomes Project data (http://www.1000genomes.org/1000-genomes-browsers) and SIRS patients

	SNP	IBS population	SIRS patients	Septic shock patients	*p* value^a^	*p* value^b^
*N*		107	263	202		
HWE (*p* value)		–	0.430	0.750		
Alleles	G	65%	63%	68%	0.684	0.306
	C	35%	37%	32%	–	–
Genotypes	GG	41.1%	40%	46%	0.481	0.229
	GC	47.7%	44%	45%	0.730	0.903
	CC	12.2%	15%	9%	0.490	0.071

*p* values were calculated by Chi-squared test: (a) differences between IBS population and septic shock patients, (b) differences between SIRS patients and septic shock patients

*SIRS* patients with systemic inflammatory response syndrome, *IBS* Iberian populations in Spain, *HWE* Hardy–Weinberg equilibrium, *IL*-*6* interleukin-6

Additional file 2: Table S2 shows the frequencies of *IL*-*6* rs1800795 polymorphism stratified according to the type of surgery. Overall, we found similar values of allelic and genotypic frequencies between cardiac and abdominal surgery compared to SIRS patients and reference population (IBS); we did not find any significant difference with healthy subjects.

### *IL*-*6* polymorphism and death in septic shock patients

We selected the recessive inheritance model (CC vs. GG/CG) for the genetic association study, because it was the model that best fit to our data.

The survival probabilities at 7, 28, and 90 days after the diagnosis of septic shock are shown in Table 3. Out of 202 patients, 111 (55%) died with a survival median of 39 days (95% CI 30.7; 47.2). Patients with *IL*-*6* rs1800795 CC genotype had lower survival probability than *IL*-*6* rs1800795 GG/CG genotype at 7 days (*p* = 0.005), 28 days (*p* = 0.009), and 90 days (*p* = 0.006).

**Table 3:** Survival probabilities at 7, 28, and 90 days (Kaplan–Meier product-limit method) for *IL*-*6* rs1800795 polymorphism in septic shock patients who underwent major cardiac or abdominal surgery

Points censoring (days)	All patients (%)	*IL*-*6* rs1800795 polymorphism		
		rs1800795 GG/CG (%)	rs1800795 CC (%)	*p* value (log-rank test)
7	86.6	88.5	68.4	0.005
28	60.7	63.2	32.9	0.009
90	14.3	15.5	0.0	0.006

*IL*-*6* interleukin-6, *p* value level of significance

 Table 4 shows the mortality risks at 7, 28, and 90 days for the *IL*-*6* rs1800795 polymorphism by Cox regression analysis. The *IL*-*6* rs1800795 CC genotype was associated with higher adjusted risk of septic shock-related death in the first 7 days [adjusted hazard ratio (aHR) 4.65; *p* = 0.002], 28 days (aHR 2.50; *p* = 0.006), and 90 days (aHR 2.28; *p* = 0.006) than *IL*-*6* rs1800795 GG/GC genotype.

**Table 4:** Risk of death regarding *IL*-*6* rs1800795 polymorphism in septic shock patients who underwent major cardiac or abdominal surgery

	Univariate			Multivariate		
	HR	95% CI	*p* value	aHR	95% CI	*p* value*
The first 7 days						
rs1800795 CC	3.42	1.36; 8.85	*0.009*	4.6	1.8; 12.1	*0.002*
Cardiac surgery				7.9	0.9; 71.1	0.064
Obesity				2.4	0.9; 6.3	0.069
Alcoholism				3.6	1.1; 11.1	*0.028*
APACHE-II score				1.1	1.1; 1.2	*0.000*
Peritonitis				11.5	1.4; 97.4	*0.025*
The first 28 days						
rs1800795 CC	2.28	1.20; 4.34	*0.012*	2.5	1.3; 4.8	*0.006*
Alcoholism				1.8	0.8; 3.7	0.135
Emergency surgery				1.9	1.0; 3.6	*0.050*
APACHE-II score				1.0	1.0; 1.1	0.052
Peritonitis				2.3	1.3; 3.9	*0.003*
Heart disease				2.0	1.2; 3.4	*0.005*
The first 90 days						
rs1800795 CC	2.20	1.23; 3.96	*0.008*	2.3	1.3; 4.1	*0.006*
Emergency surgery				1.8	1.2; 2.9	*0.008*
Peritonitis				1.9	1.2; 2.9	*0.005*
Heart disease				1.7	1.1; 2.6	*0.010*
Liver disease				2.0	0.9; 4.7	0.107

*IL*-*6* interleukin-6, *HR* hazard ratio, *aHR* adjusted hazard ratio, *95% CI* 95% confidence interval, *p* value level of significance

* *p* values were calculated by Cox regression adjusting for the most important clinical and epidemiological characteristics (see “Statistical analysis” section). Statistically significant differences are shown in italics (*p* < 0.05)

 Additional file 3: Table S3 shows the mortality risks at 7, 28, and 90 days according to *IL*-*6* rs1800795 polymorphism and stratified by type of surgery. The *IL*-*6* rs1800795 CC carrier who underwent cardiac surgery had higher risk of septic shock-related death during the first 7 days (aHR 18.39; *p* = 0.001) and 28 days (aHR 6.1; *p* = 0.025) than *IL*-*6* rs1800795 GG/GC carrier, whereas *IL*-*6* rs1800795 CC carrier who underwent abdominal surgery had higher risk of septic shock-related death during all follow-up (90 days) (aHR 1.98; *p* = 0.050) than *IL*-*6* rs1800795 GG/GC carrier.

## Discussion

To our knowledge, we described for the first time of the relationship between *IL*-*6* rs1800795 polymorphism and risk of septic shock-related death in European patients who underwent major cardiac or abdominal surgery.

The genetic variation at cytokine genes may influence the risk of sepsis and death [15]. Among these genetic variations, the SNPs at *IL*-*6* promoter are important members which might be associated with sepsis risk and death [17]. In our study, patients with *IL*-*6* rs1800795 CC genotype had higher risk of septic shock-related death, suggesting that *IL*-*6* rs1800795 polymorphism may play a major role in pathogenesis of septic shock. We analyzed allelic and genotypic frequencies of *IL*-*6* rs1800795 polymorphism between groups of patients: septic shock patients, SIRS patients, and healthy people (IBS). No significant differences were found, indicating that our cohort did not have any significant bias regarding the distribution of *IL*-*6* polymorphism.

The *IL*-*6* rs1800795 SNP has been previously associated with sepsis and death, but with discrepant results [17–21]. In European population, the rs1800795 GG genotype was associated with protection against the development of septic shock in patients with pneumococcal community-acquired pneumonia [17] and lower mortality [17, 21]. In Greek population, the rs1800795 GG genotype did not show any association with severe sepsis and mortality [18]. In Asian population, the carriers of rs1800795 C allele had higher risk of septic shock, but not higher risk of death [19]. In a recent meta-analysis, Gao et al. [20] found a statistically significant association between rs1800795 CC genotype and sepsis-related mortality (CC vs. GC/GG: OR 1.92, *p* = 0.03), which disappeared after Bonferroni correction. In this regard, it should be taken into account that only six studies, four of them with limited sample size (*n* < 60), were included for meta-analysis about sepsis-related mortality. They concluded that current evidence does not support a direct effect of rs1800795 polymorphism on the risk of sepsis and more investigations would be needed to evaluate the effect of this polymorphism on sepsis mortality.

IL-6 is a proinflammatory cytokine which plays a vital role in the regulation of host immune response in sepsis, and elevated expression of IL-6 is associated with the development of severe sepsis and mortality [11–14]. This association may be mediated through a variable IL-6 expression encoded by the host. The G to C polymorphism at position-174 of the *IL*-*6* gene (rs1800795) causes differential activity in promoter constructs which up-regulates *IL*-*6* gene transcription and promotes higher circulating levels of IL-6 in individuals that carry rs1800795 G allele [16]. However, Tischendorf et al. observed a quantitative trait locus effect, IL-6 serum concentrations were highest in patients with the rs1800795 GG genotype, followed by CG genotype, and lowest in individuals with CC genotype, but a high ex vivo secretion after LPS stimulation in rs1800795 C carriers was found [26]. These discrepancies among studies (including our study) might be due to the fact that rs1800795 polymorphism is not the causal mutation, or it is not uniquely responsible. Thus, it is possible that the effect rs1800795 SNP may also be due to other SNPs at *IL*-*6* promoter, which are in high linkage disequilibrium with rs1800795 polymorphism forming a haplotype [27, 28]. In our study, plasma IL-6 values could be very helpful and convincing to reinforce the hypothesis that the observed effect of the *IL*-*6* rs1800795 polymorphism is a real biological result. However, IL-6 measurements were not available in this study. As an alternative, a downstream biological measurement such as the white blood cell count, C reactive protein, and procalcitonin was analyzed, but we did not find any significant relationship of *IL*-*6* rs1800795 polymorphism with these markers (data not shown).

We also performed an in silico analysis for evaluating the possible functional implication of rs1800795 polymorphism by using rSNABase (http://rsnp.psych.ac.cn/) [29]. This type of analysis allows to study whether the variant is located within regulatory regions and has a possible transcriptional regulatory effect. We found that *IL*-*6* rs1800795 polymorphism, located within the promoter of *IL*-*6*, could be part of RNA-binding protein site and could be involved in RNA-binding protein-mediated post-transcriptional regulation. Moreover, etiology and pathology of sepsis are complex, and the rs1800795 polymorphism may act synergistically with other genetic factors [15], which could be contributing to the risk of death in septic shock. These factors may be SNPs in different type of interleukins, which could play different roles in development of sepsis and clinical outcome [15].

In our study, the association between rs1800795 polymorphism and septic shock-related death was also found when the population was stratified by the type of surgery. However, patients with cardiac surgery showed a significant risk of septic shock-related death in the first 7 and 28 days, whereas patients who underwent abdominal surgery had a significant risk during all follow-up (90 days). These different patterns may be intrinsic to each type of surgery, but we should not rule out the impact of the low sample size of surgery groups. Regarding the association found for cardiac surgery subgroup, the extracorporeal circulation and the surgical injury itself produce complex inflammatory responses which can lead to varying degrees of ischemia–reperfusion injury and/or systemic inflammatory response [30]. Under these circumstances, the role of *IL*-*6* rs1800795 polymorphism might be enhanced and this could justify the fact that we found a higher risk of death during the first 4 weeks of follow-up. Moreover, the association found for abdominal surgery subgroup may be due to a faster development of sepsis than in patients who underwent cardiac surgery, because an intra-abdominal infection usually occurs in the first days of post-surgical intervention [4]. In this case, the influence of the *IL*-*6* rs1800795 polymorphism on the development of septic shock-related death seems to be maintained over time, but the effect is significant when the follow-up of patients reaches 90 days.

The attributable fatality rate of septic shock is high but has significantly dropped in last two decades due to the combination of anti-infective treatments and aggressive organ failure supports [4]. However, patients become exposed to ICU-acquired complications that significantly impact on their prognosis [31, 32]. In a recent article, Daviaud et al. [33] reported that early deaths are mainly attributable to intractable multiple organ failure related to the primary infection and late deaths are related to ICU-acquired complications such as nosocomial infections and mesenteric ischemia. In our study, besides the *IL*-*6* rs1800795 polymorphism, we found a number of factors that were significantly associated with death. APACHE-II score was the most relevant factor associated with the very early mortality, but this factor disappears in favor of others like emergency surgery, peritonitis, and heart disease in the late mortality. Moreover, we included in the analysis a high number of known prognostic factors, but we cannot exclude that other variables not recorded in our study could be influencing the clinical outcome.

Finally, several limitations should be taken into account for the correct interpretation of the results. Firstly, this report has a retrospective design and the sample size was relatively small, which could limit the achievement of statistically significant values between rs1800795 polymorphism and death, especially when we performed the analysis stratified by the type of surgery (abdominal or cardiac surgery). Besides, the limited sample size might increase the risk of false positive. However, we control homogeneity by only including patients with septic shock, without mixing different stages of disease. Secondly, differences in COPD were found when patients were stratified by rs1800795 genotype, but COPD was taken into account in the multivariate analysis. Thirdly, we used multiple points of censoring for death, which may cause problems of multiple comparisons. However, here is a considerable controversy about adjusting the “*p* value” after multiple tests on clinical-orientated studies [34, 35]. In our study, we had a hypothesis supported by theory and previous reports in septic patients. Therefore, we were not literally doing a random search of a meaningful result, and our results should not be affected by the fact of carrying out a high number of statistical tests.

## Conclusions

In conclusion, the presence of *IL*-*6* rs1800795 CC genotype was associated with higher risk of septic shock-related death in patients who underwent major cardiac or abdominal surgery. The *IL*-*6* rs1800795 genotype could allow for a precision approach to the management of septic shock-related death risk. Further analysis involving large numbers of patients in independent cohorts is needed to corroborate these associations.

## Additional files

**Additional file 1: Table S1.** Summary of epidemiological and clinical characteristics of septic shock patients according to type of surgery.

**Additional file 2: Table S2.** Frequencies of alleles and genotypes for *IL-6* rs1800795 polymorphism in septic shock patients compared to Iberian populations in Spain from 1000 Genomes Project data (http://www.1000genomes.org/1000-genomes-browsers) and SIRS patients, according to type of surgery.

**Additional file 3: Table S3.** Adjusted risk of death regarding *IL-6* rs1800795 polymorphism in septic shock patients who underwent major cardiac or abdominal surgery.

## Abbreviations

IL-6: interleukin-6
JAK–STAT: Janus kinase/signal transducer and activator of transcription
SOCS3: suppressor of cytokine signaling-3
SNP: single nucleotide polymorphism
SOFA: Sequential Organ Failure Assessment
APACHE: Acute Physiology and Chronic Health Evaluation
COPD: chronic obstructive pulmonary disease
HWE: Hardy–Weinberg equilibrium
IBS: Iberian populations in Spain
aHR: adjusted hazard ratio
