# Supplementary material for: IL-6 rs1800795 polymorphism is associated with septic shock-related death in patients who underwent major surgery: a preliminary retrospective study
Source: Ann Intensive Care. 2017 Feb 28;7:22. doi: 10.1186/s13613-017-0247-8 (PMC5331026; doi:10.1186/s13613-017-0247-8)
Supplement: Supplementary file 1 — Additional file 1: Table S1. Summary of epidemiological and clinical characteristics of septic shock patients according to type of surgery. [file 13613_2017_247_MOESM1_ESM.docx]

**Additional file 1: Table S1**. Summary of epidemiological and clinical characteristics of septic shock patients according to type of surgery.

| **Characteristics** | **Cardiac surgery** | **Abdominal surgery** | **p-value (*)** |
| --- | --- | --- | --- |
| No. patients | 81 | 121 |  |
| Gender (male) | 53 (65.4%) | 75 (62.0%) | 0.616 |
| Age (years) | 72 (16) | 73.5 (19) | 0.861 |
| Emergency surgery (versus scheduled) | 29 (35.8%) | 99 (81.8%) | 0.618 |
| **Prior or pre-existing conditions** |  |  |  |
| Smoker | 14 (17.3%) | 22 (18.2%) | 0.870 |
| Alcoholism | 5 (6.2%) | 10 (8.3%) | 0.578 |
| Obesity | 17 (21.0%) | 13 (10.7%) | **0.045** |
| Diabetes | 14 (17.3%) | 12 (9.9%) | 0.125 |
| Heart disease | 56 (69.1%) | 36 (29.8%) | **<0.001** |
| Chronic obstructive pulmonary disease | 15 (18.5%) | 20 (16.5%) | 0.714 |
| Hypertension | 53 (65.4%) | 58 (47.9%) | **0.014** |
| Chronic kidney disease | 12 (14.8%) | 18 (14.9%) | 0.990 |
| Cancer | 8 (9.9%) | 39 (32.2%) | **<0.001** |
| Liver disease | 2 (2.5%) | 7 (5.8%) | 0.263 |
| **Severity indexes** |  |  |  |
| SOFA score | 9 (4) | 8 (3) | **0.030** |
| APACHE II score | 15 (6) | 17 (7) | **0.032** |
| **Infection** |  |  |  |
| Gram-positive | 46 (56.8%) | 53 (43.8%) | 0.070 |
| Gram-negative | 49 (60.5%) | 57 (47.1%) | 0.062 |
| Fungus | 14 (17.3%) | 25 (20.7%) | 0.551 |
| Catheter bacteraemia | 43 (53.1%) | 25 (20.7%) | **<0.001** |
| Surgical site infection | 16 (19.8%) | 32 (26.4%) | 0.273 |
| Urinary tract infection | 11 (13.6%) | 13 (10.7%) | 0.541 |
| Endocarditis | 10 (12.3%) | 0 (0.0%) | **<0.001** |
| Peritonitis | 0 (0.0%) | 95 (78.5%) | **<0.001** |
| Pneumonia | 58 (71.6%) | 37 (30.6%) | **<0.001** |

Values are expressed as median (interquartile range) and absolute count (percentage).

(*), P-values were calculated by Chi-square test or Fisher's exact test for categorical variables and Mann-Whitney test for continuous variables. Statistically significant differences are shown in bold.

Note that patients may have had more than one organism cultured.

**Abbreviations**: SOFA, sequential organ failure assessment; APACHE, acute physiology and chronic health evaluation.
