# Supplementary material for: IL-6 rs1800795 polymorphism is associated with septic shock-related death in patients who underwent major surgery: a preliminary retrospective study
Source: Ann Intensive Care. 2017 Feb 28;7:22. doi: 10.1186/s13613-017-0247-8 (PMC5331026; doi:10.1186/s13613-017-0247-8)
Supplement: Supplementary file 2 — Additional file 2: Table S2. Frequencies of alleles and genotypes for IL-6 rs1800795 polymorphism in septic shock patients compared to Iberian populations in Spain from 1000 Genomes Project data (http://www.1000genomes.org/1000-genomes-browsers) and SIRS patients, according to type of surgery. [file 13613_2017_247_MOESM2_ESM.docx]

**Additional file 2: Table S2.** Frequencies of alleles and genotypes for *IL-6* rs1800795 polymorphism in septic shock patients compared to Iberian populations in Spain from 1000 Genomes Project data (<http://www.1000genomes.org/1000-genomes-browsers>) and SIRS patients, according to type of surgery.

| **Cardiac surgery** | **SNP** | **IBS population** | **SIRS patients** | **Septic shock patients** | **p-value ^(a)^** | **p-value ^(b)^** |
| --- | --- | --- | --- | --- | --- | --- |
| **No.** |  | 107 | 152 | 81 |  |  |
| **HWE (p-value)** |  | - | 0.120 | 0.450 |  |  |
| **Alleles** | G | 65% | 62% | 69% | 0.674 | 0.358 |
|  | C | 35% | 38% | 31% | - | - |
| **Genotypes** | GG | 41.1% | 41% | 46% | 0.610 | 0.551 |
|  | GC | 47.7% | 41% | 47% | 0.958 | 0.458 |
|  | CC | 12.2% | 18% | 7% | 0.351 | **0.036** |
|  |  |  |  |  |  |  |
| **Abdominal surgery** | **SNP** | **IBS population** | **SIRS patients** | **Septic shock patients** | **p-value ^(a)^** | **p-value ^(b)^** |
| **No.** |  | 107 | 112 | 121 |  |  |
| **HWE (p-value)** |  | - | 0.680 | 0.840 |  |  |
| **Alleles** | G | 65% | 64% | 68% | 0.735 | 0.613 |
|  | C | 35% | 36% | 32% | - | - |
| **Genotypes** | GG | 41.1% | 39% | 46% | 0.541 | 0.347 |
|  | GC | 47.7% | 49% | 43% | 0.563 | 0.431 |
|  | CC | 12.2% | 12% | 11% | 0.940 | 0.973 |

P-values were calculated by Chi-squared test: (a), differences between IBS population and septic shock patients; (b), differences between SIRS patients and septic shock patients. Statistically significant differences are shown in bold.

Abbreviations: SIRS, patients with systemic inflammatory response syndrome; HWE, Hardy Weinberg Equilibrium; IBS, Iberian population in Spain; IL-6, interleukin.
