# Supplementary material for: IL-6 rs1800795 polymorphism is associated with septic shock-related death in patients who underwent major surgery: a preliminary retrospective study
Source: Ann Intensive Care. 2017 Feb 28;7:22. doi: 10.1186/s13613-017-0247-8 (PMC5331026; doi:10.1186/s13613-017-0247-8)
Supplement: Supplementary file 3 — Additional file 3: Table S3. Adjusted risk of death regarding IL-6 rs1800795 polymorphism in septic shock patients who underwent major cardiac or abdominal surgery. [file 13613_2017_247_MOESM3_ESM.docx]

**Additional file 3: Table S3.** Adjusted risk of death regarding *IL-6* rs1800795 polymorphism in septic shock patients who underwent major cardiac or abdominal surgery.

|  | **Univariate** | | | **Multivariate** | | |
| --- | --- | --- | --- | --- | --- | --- |
| **Cardiac surgery** | **HR** | **95%CI** | **p-value** | **aHR** | **95%CI** | **p-value (*)** |
| **The first 7 days** |  |  |  |  |  |  |
| rs1800795 CC | 8.99 | 2.14; 37.76 | **0.003** | 18.39 | 3.31; 102.1 | **0.001** |
| **The first 28 days** |  |  |  |  |  |  |
| rs1800795 CC | 4.27 | 1.20; 15.12 | **0.024** | 6.1 | 1.25; 29.8 | **0.025** |
| **The first 90 days** |  |  |  |  |  |  |
| rs1800795 CC | 4.27 | 1.20; 15.12 | **0.024** | 3.48 | 0.77; 15.64 | 0.103 |
|  | **Univariate** | | | **Multivariate** | | |
| **Abdominal surgery** | **HR** | **95%CI** | **p-value** | **aHR** | **95%CI** | **p-value (*)** |
| **The first 7 days** |  |  |  |  |  |  |
| rs1800795 CC | 1.91 | 0.55; 6.65 | 0.309 | 2.78 | 0.73; 10.56 | 0.133 |
| **The first 28 days** |  |  |  |  |  |  |
| rs1800795 CC | 1.63 | 0.77; 3.46 | 0.202 | 1.89 | 0.88; 4.04 | 0.101 |
| **The first 90 days** |  |  |  |  |  |  |
| rs1800795 CC | 1.69 | 0.86; 3.30 | 0.126 | 1.98 | 1.00; 3.93 | **0.050** |

(*) P-values were calculated by Cox regression adjusting for the most important clinical and epidemiological characteristics (see **statistical analysis** section).

**Abbreviations**: HR, hazard ratio; aHR, adjusted hazard ratio; 95%CI, 95% confidence interval; p-value, level of significance.
